# Family Caregivers of People with Dementia Associate with Poor Health-Related Quality of Life: A Nationwide Population-Based Study

**DOI:** 10.3390/ijerph192316252

**Published:** 2022-12-05

**Authors:** Ji Hye Shin, Ji Hyun Kim

**Affiliations:** Department of Neurology, Korea University Guro Hospital, Seoul 08308, Republic of Korea

**Keywords:** people with dementia, family caregivers, health-related quality of life

## Abstract

Despite the growing awareness of poor health-related quality of life (HRQoL) in family caregivers of people with dementia (PWD), their relationship has rarely been explored with population-based samples. The current cross-sectional study aimed to determine the detrimental impact of informal dementia caregiving on HRQoL by using nationally representative population-based samples from the Korean Community Health Survey. Demographics, socioeconomic, and physical and mental health-related characteristics as well as HRQoL measured by the Korean version of the European Quality of Life Questionnaire Five Dimension (EQ-5D) were compared between 9563 family caregivers of PWD and 186,165 noncaregivers. Caregivers had lower index scores and higher frequency of some/extreme problems in all five dimensions of the EQ-5D compared with noncaregivers. Logistic regression adjusting for potential confounding factors found that caregivers had a higher frequency of poor HRQoL (lowest quartile of EQ-5D index) than noncaregivers (adjusted odds ratio [95% confidence interval] = 1.46 [1.39–1.53]). Compared to noncaregivers, caregivers had a higher frequency of some/extreme problems in each dimension of the EQ-5D: mobility (1.30 [1.21–1.40]), self-care (1.62 [1.46–1.80]), usual activity (1.39 [1.29–1.51]), pain/discomfort (1.37 [1.31–1.45]), and anxiety/depression (1.51 [1.42–1.61]). A one-to-one propensity score matching analysis confirmed that poor HRQoL was more frequently found in caregivers compared to noncaregivers (1.38 [1.29–1.48]). Our results indicated that family caregivers of PWD are significantly associated with overall poor HRQoL, underscoring the detrimental impact of informal dementia caregiving on HRQoL. Given the high frequency of poor HRQoL in dementia caregivers and the important recognition of its serious consequences on physical and mental health, clinicians should take into consideration efficient interventions to improve health and HRQoL for family caregivers of PWD.

## 1. Introduction

The worldwide number of people with dementia (PWD) in 2020 was estimated at about 55 million [1]. The number of PWD in South Korea is currently estimated at approximately 0.8 million, with the number anticipated to escalate to 3.02 million by 2050 [2]. Dementia leads to a global public health problem and is a major cause of mortality and morbidity [3]. The annual cost of dementia caregiving was calculated to be approximately 13 billion USD in Korea, in 2019 [4], which is expected to increase steadily as the disease progresses [5]. Based on the disease burden measured by disability-adjusted life years, dementia is one of the most burdensome disorders, not only to dementia patients but also to their informal or family caregivers [3]. The costs for both professional and informal (unpaid) caregiving increase significantly as the patients become more dependent on their caregivers [6].

Dementia is an irreversible and chronic progressive disease that mostly affects the elderly, characterized by an ongoing decline in physical, cognitive, and social functioning [3]. Moreover, PWD manifests behavioral and emotional symptoms such as physical/verbal aggression, anxiety/depression, sleep problems, and resistance to care [7]. Caregiving stands for attending to the other person’s well-being and health needs and commonly involves supporting their activities of daily living [8]. Caregiving of PWD usually takes place over several years to decades and is largely shouldered by the family including siblings, spouses, and children. In the U.S., in 2019, 16.3 million family caregivers spent an average of 1139 h per year or 21.9 h per week caregiving for PWD [3].

Caregiving of PWD is physically, emotionally, and financially demanding, and caregivers are very likely to be angered, burdened by responsibility, isolated, and finally depressed. About 30–40% of family caregivers of PWD reported depressive symptoms, compared to 5–17% of noncaregivers of the same ages [9,10]. The demands of caregiving could give rise to a deterioration in caregivers’ overall health [11]. Evidence from meta-analyses suggests that the overall health of the family caregivers is generally poor, which is intimately related to their depression and anxiety, patients’ behavioral/emotional problems, and emotional and physical stressors associated with dementia [12,13,14,15]. Although there is no single accepted theoretical model underlying the conceptualization and assessment of health outcomes in family caregivers of PWD, Vitaliano et al. [15] proposed a theoretical model for the physical health effects of caregiving as the consequence of exposure to stress, which may be moderated by vulnerabilities and resources and mediated by psychosocial distress and health behaviors. In a systematic review of determinant models of caregiver burden and mental health, van der Lee et al. [16] suggested that the behavioral problems of PWD, caregivers’ coping and personality traits, and competence are the most consistent determinants of caregiver burden, depression and mental health of dementia caregivers.

Family caregivers of PWD reported a higher frequency of behavioral problems (15% vs. 4%), mental health problems (41% vs. 16%), the strain of caregiving (22% vs. 11%), and a high level of burden (46% vs. 38%) than family caregivers of people without dementia [3]. Moreover, it has been shown that compared to noncaregivers, caregivers of PWD exhibited poorer cognitive performance in verbal memory, attention, and frontal executive function [17,18,19,20,21,22,23], which may be potentially attributed to their chronic stress related to dementia caregiving and psychological factors [24]. Poor sleep quality and sleep disturbances that have been well documented in family caregivers of PWD might also be implicated in their cognitive impairments [19,25,26,27,28,29,30,31,32,33].

Health-related quality of life (HRQoL) is a general concept reflecting the person’s psychological status, physical health, social relationships, independence level, and environmental supports [34]. HRQoL assesses the influence of disability and mental and physical disorders on a person’s well-being. Assessing HRQoL is a critical challenge confronting the economic evaluation of psychosocial and pharmacological interventions in the field of dementia research [35,36]. Previous studies have repeatedly shown that dementia caregivers experienced poorer HRQoL in comparison with noncaregivers or the general population [37,38,39,40,41] and that caregivers’ HRQoL was closely linked to their depressive symptoms, caregiver burden, and dementia severity and progression [38,42,43,44,45,46,47,48,49].

Despite the increasing awareness of poor HRQoL in family caregivers of PWD, their connection has rarely been explored with population-based samples [40,41]. The current study aimed at evaluating the detrimental impact of informal dementia caregiving on HRQoL by using nationally representative population-based samples. The Korean version of the European Quality of Life Questionnaire Five Dimensions (EQ-5D) was used to measure HRQoL, as it offers a comprehensive assessment of the person’s health state in emotional and physical dimensions [50,51]. Assessing the HRQoL of family caregivers of PWD would determine caregiver burden and help clinicians to establish appropriate management strategies to enhance their HRQoL.

## 2. Materials and Methods

### 2.1. Study Population

For the current study, we obtained data from the 2018 Korean Community Health Survey (KCHS), a nationwide cross-sectional survey conducted annually since 2008 by the Korean government. This survey investigates the trends of disease prevalence and morbidity, physical and mental health-related behaviors, and personal lifestyle in adults aged 19 or more years [52]. The sample size is 900 participants in each of the 254 community health units located in 17 metropolitan cities and provinces, and the expected total number of participants is 228,600. The KCHS utilized a two-stage sampling procedure to assure a better representation of the general population, as described in detail elsewhere [33,53]. A total of 32,612 out of 228,340 participants were ruled out because they did not fill in the survey variables listed in Table 1 and Table 2, and consequently, 195,728 participants were eventually included in the statistical analysis.

All data in the KCHS are available publicly and totally anonymized prior to public release. Thus the current study does not require separate ethics approval from the local ethics committee.

### 2.2. Independent Variable: Dementia Caregiving

Participants were asked whether he/she (spouse, parents, children, siblings, relative) provides care for a person who has been diagnosed with dementia by a clinician. The response was either yes (family caregivers) or no (noncaregivers).

### 2.3. Dependent Variable of Main Interest: Health-Related Quality of Life

HRQoL was evaluated with the Korean version of EQ-5D, an extensively used, self-report instrument to assess HRQoL [50,51]. It comprises five dimensions: mobility, self-care, usual activity, pain/discomfort, and anxiety/depression. Each dimension is rated by asking a question with three levels of response: no problem, some problems, or extreme problems. The EQ-5D index was further computed to represent the overall health state by utilizing the Korean version of the value set that was derived from a representative national sample of the Korean general population, using the time trade-off method [54]. It ranges from −0.171 to 1, with lower values representing the overall poorer health state and 1 the best health state [54]. For statistical analysis, HRQoL was further divided into quartiles based on the EQ-5D index: first (lowest) quartile as poor, second as fair, third as good, and fourth (highest) as very good. The first (lowest) quartile was regarded as a marker of poor HRQoL.

### 2.4. Covariates

Demographics included age, sex, and body mass index. Socioeconomic variables included residential area (rural or urban), education level (elementary school or lower, middle school, high school, or college or higher), marital status (living with spouse or living alone), employment status (employed or unemployed), and household income level (low, middle-low, middle-high, or high).

Physical health-related variables included smoking status (non/ex-smoker or current smoker), risky drinking (≥12 drinking episodes that consumed ≥ 5 alcoholic glasses during the past year) [55], regular exercise (≥30 min of walking for ≥5 days a week) [56], and presence of diabetes mellitus and hypertension.

Mental health-related variables included perceived stress (rare/mild as no or moderate/severe as yes), perceived health status (good, moderate, or bad), subjective cognitive decline, depression, and sleep quality. Subjective cognitive decline was assessed based on the Behavioral Risk Factor Surveillance System [57]. The response to the following question, “Have you experienced recurrent or worsening of memory impairment or confusion during the past year?” was either yes or no. Depressive symptoms were determined by adopting the Korean version of the Patient Health Questionnaire-9 (PHQ-9) [58,59]. The presence of depression was classified into yes (global PHQ-9 score ≥ 10) or no (global PHQ-9 score ≤ 9). Sleep quality was evaluated by adopting the Korean version of the Pittsburgh Sleep Quality Index (PSQI) [60,61], which provides high test-retest reliability and validity and proved to be a reliable indicator for differentiating poor sleepers from good sleepers [62]. Good sleep quality was indicated by a global PSQI score ≤ 5, and poor sleep quality by a global PSQI score ≥ 6 [63].

### 2.5. Statistics

Demographics, socioeconomic characteristics, physical and mental health-related variables, and HRQoL (EQ-5D) were compared between family caregivers of PWD and noncaregivers using the Chi-square test or Student’s *t*-test, where appropriate.

Multivariable logistic regression was performed to determine the association between dementia caregiving and poor HRQoL (lowest quartile of the EQ-5D index). The statistical models were controlled for age, sex, and body mass index (Model 1), further controlled for socioeconomic variables (education level, residential area, marital status, household income, and employment status) (Model 2), and further controlled for physical health-related variables (smoking, risky drinking, regular exercise, diabetes mellitus, and hypertension) and mental health-related variables (perceived stress, perceived health status, depression, subjective cognitive decline, and sleep quality) (Model 3). Moreover, multivariable logistic regression was repeated on each of the five EQ-5D dimensions to determine the association between caregiving of PWD and having problems in each dimension (no problem vs. some/extreme problems).

Given the differences in sample size and baseline characteristics between caregivers and noncaregivers, the propensity score matching method was further performed to control for confounding. Propensity scores were calculated for each participant using a binary logistic regression involving the demographics and socioeconomic variables. Caregivers were then matched with noncaregivers who had similar probabilities of having been caregivers, according to their propensity scores. A one-to-one propensity score matching was conducted with the nearest neighbor algorithm using calipers of width equal to 0.2 of the standard deviation of the logit of the propensity score. Group comparisons and multivariable logistic regression were repeated using the propensity-matched sample. Results were presented as adjusted odds ratios (ORs) and 95% confidence intervals (CIs). Statistical analyses were performed by using the Statistical Package for Social Sciences (version 26.0; IBM, Armonk, NY, USA).

## 3. Results

Demographics, socioeconomic characteristics, and physical and mental health-related variables of the family caregivers of PWD (*n* = 9563) and noncaregivers (*n* = 186,165) are summarized in Table 1. Caregivers versus noncaregivers were older (*p* < 0.001) and more likely to reside in rural areas (*p* = 0.018), have less college education (*p* < 0.001), live with their spouses (*p* < 0.001), and have high household income level (*p* < 0.001). There were no significant differences in sex, body mass index, employment status, and smoking status between the groups. Risky drinking (28.1% vs. 26.0%) and regular exercise (45.9% vs. 43.9%) were more frequently found in noncaregivers relative to caregivers (both *p* < 0.001). Caregivers versus noncaregivers had a higher frequency of diabetes mellitus (12.6% vs. 10.4%), hypertension (28.4% vs. 26.3%), subjective cognitive decline (23.9% vs. 16.0%), perceived stress (28.4% vs. 22.8%), bad perceived health status (21.7% vs. 17.4%), depression (5.1% vs. 2.9%), and poor sleep quality (36.1% vs. 29.1%) (all *p* < 0.001). 

Comparisons of the EQ-5D index score and five dimensions between the caregivers and noncaregivers are presented in Table 2. The EQ-5D index score was lower in caregivers compared to noncaregivers (0.92 ± 0.14 vs. 0.95 ± 0.10, *p* < 0.001). As expected, poor HRQoL was more frequently observed in caregivers compared to noncaregivers (41.6% vs. 30.8%, *p* < 0.001). Caregivers had a higher frequency of some and extreme problems than noncaregivers in all five dimensions: mobility (21.7% vs. 12.2%), self-care (8.5% vs. 3.9%), usual activity (16.8% vs. 8.6%), pain/discomfort (33.6% vs. 24.7%), and anxiety/depression (17.4% vs. 10.5%) (all *p* < 0.001). 

Table 3 presents the associations between dementia caregiving and poor HRQoL after controlling for demographics (Model 1), demographics and socioeconomic variables (Model 2), and demographics, socioeconomic, and mental and physical health-related variables (Model 3). Poor HRQoL was more frequently found in family caregivers compared to noncaregivers (Model 1: OR 1.52, 95% CI 1.45–1.58; Model 2: OR 1.66, 95% CI 1.59–1.74; Model 3: OR 1.46, 95% CI 1.39–1.53). In the fully adjusted model (Model 3) evaluating separately each of the five EQ-5D dimensions, caregivers versus noncaregivers had a higher frequency of experiencing some/extreme problems in mobility (OR 1.30, 95% CI 1.21–1.40), self-care (OR 1.62, 95% CI 1.46–1.80), usual activity (OR 1.39, 95% CI 1.29–1.51), pain/discomfort (OR 1.37, 95% CI 1.31–1.45), and anxiety/depression (OR 1.51, 95% CI 1.42–1.61).

A one-to-one propensity score matching identified 9563 caregivers and 9563 noncaregivers matched for demographics and socioeconomic variables (Table 4). Similar to the unmatched analysis, caregivers versus noncaregivers had a higher frequency of diabetes mellitus (12.6% vs. 11.5%, *p* = 0.012), perceived stress (28.4% vs. 22.0%), bad perceived health status (21.7% vs. 18.5%), subjective cognitive decline (23.9% vs. 18.1%), depression (5.1% vs. 2.6%), and poor sleep quality (36.1% vs. 29.1%) (all *p* < 0.001). The EQ-5D index score was lower in caregivers compared to noncaregivers (0.92 ± 0.14 vs. 0.95 ± 0.10, *p* < 0.001). As expected, poor HRQoL was more frequently observed in caregivers compared to noncaregivers (41.6% vs. 31.8%, *p* < 0.001). Multivariable logistic regression controlling for physical and mental health-related variables showed that poor HRQoL was more frequently found in family caregivers compared to noncaregivers (OR 1.38, 95% CI 1.29–1.48).

## 4. Discussion

The current study is, to our knowledge, the first to show a significant association between family caregivers of PWD and poor HRQoL by using a nationally representative population-based survey in Korea. The main results are that family caregivers of PWD reported lower EQ-5D index scores and more some/extreme problems in all five dimensions compared with noncaregivers, even after controlling for many potential confounders. In good agreement with previous investigations, our results indicate that dementia caregivers have poorer overall HRQoL than noncaregivers.

Previous case-control studies repeatedly demonstrated a significant relationship between dementia caregiving and poor HRQoL. Compared to a normative sample from the U.S. general population, the 2477 dementia caregivers had lower physical and mental summary scores, as assessed by the Short Form (SF-12) [37]. The SF-12 mental functioning scores of the caregivers were inversely correlated with depressive symptoms and disruptive behaviors of the patients and the hours of caregiving, implying that the caregiving burden has considerable impacts on the HRQoL of the family caregivers of PWD [37]. In a study conducted in Greece, 155 family caregivers of Alzheimer’s disease had lower HRQoL scores in most dimensions of the Short Form 36 Health Survey (SF-36), a widely used questionnaire for use in health policy evaluation, clinical research, and population-based survey [38,64]. In addition, the depression scale score was inversely correlated with all SF-36 dimension scores, suggesting that dementia caregiving leads to poorer HRQoL, which strongly affects depression symptoms in the family caregivers of dementia patients [38]. In a longitudinally designed study, 236 family caregivers of patients with Alzheimer’s disease had poorer HRQoL than the sex- and age-matched general population of Finland [39], as assessed by the 15D instrument that is a generic, comprehensive, 15-dimensional, standardized measure of HRQoL [65]. This difference in HRQoL between caregivers and their counterparts remained significant throughout the 3 years of follow-up period [39]. 

In the current study, logistic regression adjusting for confounding factors such as socioeconomic and physical and mental health-related characteristics provides robust support for the association between dementia caregiving and poor HRQoL. A propensity score matching analysis confirmed that poor HRQoL was more frequently found in caregivers compared to noncaregivers. Our main result of poorer HRQoL in family caregivers of PWD relative to noncaregivers aligns well with those of two currently available population-based studies. In a population-based study using the 2012/2013 National Health and Wellness Survey in Japan, compared with 53,758 noncaregivers, 1302 family caregivers had lower health utility scores as well as mental and physical component scores derived from the SF-36 questionnaire [40]. The results were replicated with a propensity matching analysis combining the 1297 caregivers and 1297 noncaregivers, corroborating that family caregivers of PWD have poorer HRQoL. Comparable findings were found in a Brazilian population-based study using the internet-based survey stratified by age and sex to ensure representation of the general adult population (aged ≥18 years) [41]. Compared to 10,644 noncaregivers, 209 family caregivers exhibited lower health utility and mental component summary scores and a trend toward significance on lower physical component summary scores, suggesting poor mental HRQoL and overall health in dementia caregivers [41]. Taken together, the results of the aforementioned studies indicate that family caregivers of PWD experienced poorer HRQoL than noncaregivers, which may be affected by caregiver burden, depressive symptoms, and dementia severity [38,42,43,44,45,46,47,48,49]. Given that dementia caregivers who underwent intervention programs such as psychoeducation [66,67,68], physical activity program [69], occupational therapy [70,71], or multicomponent intervention [72,73], had significantly better HRQoL than those without such an intervention, family caregivers of dementia patients should be comprehensively evaluated for HRQoL and targeted for efficient intervention programs to improve their HRQoL.

The deleterious impacts of family caregiving on mental and physical as well as cognitive functions have also been demonstrated in prior investigations. More specifically, the family caregivers of patients with various dementia disorders were more likely to have an increased risk of cardiovascular diseases [74,75,76], poor physical health status [13,16,24], poor psychological health status including strain, depression, and anxiety [25,77,78,79], sleep disturbances and poor sleep quality [19,25,26,27,28,29,30,31,32,33], and cognitive impairments [17,18,19,20,21,22,23]. Consistent with the aforementioned studies, the fully adjusted model of the current study disclosed that caregivers had a higher frequency of diabetes mellitus, hypertension, depression, subjective cognitive decline, and poor sleep quality relative to noncaregivers (all *p* < 0.001). In addition, caregivers reported poorer perceived health status (*p* = 0.004) and higher stress levels (*p* < 0.001) than noncaregivers, further supporting the notion that dementia caregiving is closely related to poor physical and mental health statuses as well as cognitive impairments.

Dementia is a chronic progressive disorder manifested by memory and other cognitive impairments, functional and social disabilities, and emotional disturbances, resulting in immense physical and mental health consequences for the affected individuals, their family caregivers, and society [3]. The assessment of HRQoL in PWD and their caregivers presents a significant challenge [35,36], and the instrument can be disease-specific (e.g., QoL-AD) [80,81] or generic (e.g., EQ-5D, SF-36, 15D) [50,64,65]. The EQ-5D, a nondisease-specific and standardized tool for measuring HRQoL, has been extensively utilized in the clinical and socioeconomic evaluation of healthcare interventions owing to its high responsiveness, validity, reliability, and short execution time [50,51]. It proved to be valid for diverse populations and age groups, applicable to people with various health disorders as well as healthy populations, and able to provide health patterns and utility indices. Owing to these advantages, the EQ-5D has been widely applied to various dementia research over the past 20 years, from assessing HRQoL in people with different types of dementia to estimating the burden and costs of dementia caregiving [82]. Furthermore, the EQ-5D proved to be comparable to other generic instruments in dementia studies, and thus, has been adopted as an outcome measure in population-based studies and clinical trials as well as psychosocial interventions for dementia [82]. Nevertheless, there is substantial concern and debate regarding the appropriateness and validity of the EQ-5D self-ratings for PWD, even in patients with mild dementia [82,83,84]. In the current study, any respondents who were diagnosed with dementia were excluded from the analysis, making it less likely that unreliable self-ratings of the EQ-5D have affected the results.

The strengths of the current study lie in the selection of large samples from a nationwide population-based survey and the adoption of a sampling method ensuring a good representation of the general population. Moreover, the KCHS provided information about many confounding factors that can potentially affect HRQoL, which enabled exploring the relationship between dementia caregiving and poor HRQoL following statistical adjustments. The current study has at least four limitations that should be considered before drawing conclusions. First, the cross-sectional study design precludes any firm conclusions with regard to causal relationships and the directionality of associations. Second, the self-report nature of the survey may produce recall bias of under-reporting or over-reporting. Third, the sample size of noncaregivers was approximately 20 times larger than that of caregivers, possibly skewing the results in the direction of noncaregivers. In the current study, a propensity score matching analysis was employed to reduce potential confounding effects of the sample size and baseline characteristics between the groups, confirming the results of the unmatched analysis. Last, the KCHS did not provide information on the level and type of caregiving as well as time spent in caregiving, nor did it collect information regarding the specific type of dementia (e.g., Alzheimer’s disease, frontotemporal dementia, or Lewy body dementia) and disease progression and severity. Caregiver burden and HRQoL of the family caregivers could differ according to the different forms of dementia and the progression and severity of the disease [85,86,87,88]. Thus, future longitudinal studies should provide detailed information about caregiver burden and surrounding issues including physical and mental measures, and estimate the specific contributions of dementia types and severity to the caregiver burden and HRQoL of dementia caregivers. Such research certainly helps elucidate the comprehensive impacts of dementia caregiving on the caregivers’ HRQoL and better understand the need for efficient interventions to improve their HRQoL.

## 5. Conclusions

Our results from a nationally representative population-based survey using the EQ-5D, underscore the importance of recognizing overall poor HRQoL in family caregivers of PWD. Specifically, dementia caregivers experienced a higher frequency of problems in mobility, self-care, usual activity, pain/discomfort, and anxiety/depression, compared with noncaregivers. Our results further reinforce the previous findings that dementia caregivers are frequently accompanied by physical and mental health-related comorbidities, including hypertension, diabetes mellitus, strain, cognitive impairment, depression, and poor sleep quality. For the appropriate management of dementia caregivers’ poor HRQoL, it is important to evaluate comprehensively the severity and nature of their HRQoL and surrounding issues such as caregiver burden, dementia progression and severity, and physical and mental comorbidities. Given the high frequency of poor HRQoL in dementia caregivers and the growing recognition of its serious consequences on mental and physical health, clinicians should take into consideration efficient interventions to improve health and HRQoL for family caregivers of PWD [89].

## Figures and Tables

**Table 1:** Baseline characteristics of the family caregivers of people with dementia and noncaregivers.

Variables	Family Caregivers (*n* = 9563)	Noncaregivers (*n* = 186,165)	*p* Value
Age (year)	56.0 ± 14.3	52.9 ± 17.0	<0.001
Sex			0.395
Male	4426 (46.3)	86,997 (46.7)	
Female	5137 (53.7)	99,168 (53.3)	
Body mass index (kg/m^2^)	23.7 ± 3.2	23.6 ± 3.3	0.643
Residential area			0.018
Urban	5524 (57.8)	109,801 (59.0)	
Rural	4039 (42.2)	76,364 (41.0)	
Education			<0.001
Elementary school or lower	1729 (18.2)	37,812 (20.3)	
Middle school	1276 (13.3)	21,563 (11.6)	
High school	3332 (34.8)	55,527 (29.8)	
College or higher	3226 (33.7)	71,263 (38.3)	
Marital status			<0.001
Married (living with spouse)	7634 (76.8)	128,028 (68.8)	
Living alone (never married, separated, divorced, widowed)	2217 (23.2)	58,137 (31.2)	
Employment status			0.084
Employed	6053 (63.3)	119,454 (64.2)	
Unemployed	3510 (36.7)	66,711 (35.8)	
Household income			<0.001
Low	2389 (25.0)	48,183 (25.9)	
Middle-low	2659 (27.8)	53,347 (28.7)	
Middle-high	2652 (27.7)	52,446 (28.2)	
High	1863 (19.5)	32,189 (17.2)	
Smoking			0.062
Current smoker	1659 (17.3)	33,700 (18.1)	
Non/ex-smoker	7904 (82.7)	152,465 (81.9)	
Risky drinking			<0.001
Yes	2491 (26.0)	52,373 (28.1)	
No	7072 (74.0)	133,792 (71.9)	
Regular exercise			<0.001
Yes	4198 (43.9)	85,374 (45.9)	
No	5365 (56.1)	100,791 (54.1)	
Hypertension			<0.001
Yes	2716 (28.4)	49,054 (26.3)	
No	6847 (71.6)	137,111 (73.7)	
Diabetes mellitus			<0.001
Yes	1208 (12.6)	19,375 (10.4)	
No	8355 (87.4)	166,790 (89.6)	
Perceived stress			<0.001
Yes	2719 (28.4)	42,368 (22.8)	
No	6844 (71.6)	143,797 (77.2)	
Perceived health status			<0.001
Good	2975 (31.1)	69,051 (37.1)	
Moderate	4510 (47.2)	84,769 (45.5)	
Bad	2078 (21.7)	32,345 (17.4)	
Subjective cognitive decline			<0.001
Yes	2284 (23.9)	31,495 (16.9)	
No	7279 (76.1)	154,670 (83.1)	
PHQ-9 global score	2.7 ± 3.7	2.0 ± 3.0	<0.001
Depression			<0.001
Yes (PHQ-9 score ≥ 10)	492 (5.1)	5474 (2.9)	
No (PHQ-9 score ≤ 9)	9071 (94.9)	180,691 (97.1)	
PSQI global score	5.2 ± 3.1	4.7 ± 2.8	<0.001
Good sleep quality (PSQI ≤ 5)	6112 (63.9)	131,941 (70.9)	<0.001
Poor sleep quality (PSQI ≥ 6)	3451 (36.1)	54,224 (29.1)	

Note: Data are expressed as number (percent) or mean ± standard deviation. Group comparisons were made using the Chi-square test or Student’s *t*-test. Abbreviations: PHQ-9, Patient Health Questionnaire-9; PSQI, Pittsburgh Sleep Quality Index.

**Table 2:** Comparisons of the EQ-5D index score and five dimensions between family caregivers of people with dementia and noncaregivers.

EQ-5D	Family Caregivers (*n* = 9563)	Noncaregivers (*n* = 186,165)	*p* Value
Index score	0.92 ± 0.14	0.95 ± 0.10	<0.001
Good HRQoL	5586 (58.4)	128,863 (69.2)	<0.001
Poor HRQoL	3977 (41.6)	57,302 (30.8)	
Mobility			<0.001
No problem	7492 (78.3)	163,437 (87.8)	
Some problems	1934 (20.3)	21,939 (11.8)	
Extreme problems	137 (1.4)	789 (0.4)	
Self-care			<0.001
No problem	87,536 (91.5)	178,922 (96.1)	
Some problems	692 (7.2)	6331 (3.4)	
Extreme problems	118 (1.3)	912 (0.5)	
Usual activity			<0.001
No problem	7955 (83.2)	170,097 (91.4)	
Some problems	1444 (15.1)	15,523 (8.3)	
Extreme problems	164 (1.7)	545 (0.3)	
Pain/discomfort			<0.001
No problem	6349 (66.4)	140,242 (75.3)	
Some problems	2898 (30.3)	42,626 (22.9)	
Extreme problems	316 (3.3)	3297 (1.8)	
Anxiety/depression			<0.001
No problem	7901 (82.6)	166,682 (89.5)	
Some problems	1519 (15.9)	18,486 (9.9)	
Extreme problems	143 (1.5)	997 (0.6)	

Note: Data are expressed as number (percent) or mean ± standard deviation. Group comparisons were made using the Chi-square test or Student’s *t*-test.

**Table 3:** Results of multivariable logistic regression showing significant associations between family caregivers of people with dementia and poor health-related quality of life.

	Model 1		Model 2		Model 3	
	OR	95% CI	OR	95% CI	OR	95% CI
EQ-5D index						
Good HRQoL	1	Reference	1	Reference	1	Reference
Poor HRQoL	1.52	1.45–1.58	1.66	1.59–1.74	1.46	1.39–1.53
Mobility						
No problem	1	Reference	1	Reference	1	Reference
Some/extreme problems	1.39	1.30–1.49	1.51	1.41–1.62	1.30	1.21–1.40
Self-care						
No problem	1	Reference	1	Reference	1	Reference
Some/extreme problems	1.86	1.69–2.05	1.97	1.79–2.18	1.62	1.46–1.80
Usual activity						
No problem	1	Reference	1	Reference	1	Reference
Some/extreme problems	1.52	1.42–1.63	1.66	1.54–1.78	1.39	1.29–1.51
Pain/discomfort						
No problem	1	Reference	1	Reference	1	Reference
Some/extreme problems	1.45	1.39–1.52	1.56	1.49–1.64	1.37	1.31–1.45
Anxiety/depression						
No problem		Reference	1	Reference	1	Reference
Some/extreme problems	1.75	1.65–1.85	1.88	1.78–1.99	1.51	1.42–1.61

Note: The statistical models were controlled for demographics (Model 1), further controlled for socioeconomic variables (Model 2), and further controlled for physical and mental health-related variables (Model 3). Abbreviations: CI, confidence interval; EQ-5D, European Quality of Life Questionnaire Five Dimension; OR, odds ratio.

**Table 4:** Comparisons of the physical and mental health-related variables and HRQoL (EQ-5D) between family caregivers of people with dementia and noncaregivers in propensity-score matched samples.

Variables	Family Caregivers (*n* = 9563)	Noncaregivers (*n* = 9563)	*p* Value
Smoking			0.216
Current smoker	1659 (17.3)	1621 (17.0)	
Non/ex-smoker	7904 (82.7)	7942 (83.0)	
Risky drinking			0.586
Yes	2491 (26.0)	2458 (25.7)	
No	7072 (74.0)	7105 (74.3)	
Regular exercise			0.106
Yes	4198 (43.9)	4309 (45.1)	
No	5365 (56.1)	5254 (54.9)	
Hypertension			0.065
Yes	2716 (28.4)	2832 (29.6)	
No	6847 (71.6)	6731 (70.4)	
Diabetes mellitus			0.012
Yes	1208 (12.6)	1095 (11.5)	
No	8355 (87.4)	8468 (88.5)	
Perceived stress			<0.001
Yes	2719 (28.4)	2103 (22.0)	
No	6844 (71.6)	7460 (78.0)	
Perceived health status			<0.001
Good	2975 (31.1)	3389 (35.4)	
Moderate	4510 (47.2)	4413 (46.1)	
Bad	2078 (21.7)	1761 (18.5)	
Subjective cognitive decline			<0.001
Yes	2284 (23.9)	1728 (18.1)	
No	7279 (76.1)	7835 (81.9)	
PHQ-9 global score	2.7 ± 3.7	2.0 ± 2.9	<0.001
Depression			<0.001
Yes (PHQ-9 score ≥ 10)	492 (5.1)	248 (2.6)	
No (PHQ-9 score ≤ 9)	9071 (94.9)	9315 (97.4)	
PSQI global score	5.2 ± 3.1	4.7 ± 2.8	<0.001
Good sleep quality (PSQI ≤ 5)	6112 (63.9)	6782 (70.9)	<0.001
Poor sleep quality (PSQI ≥ 6)	3451 (36.1)	2781 (29.1)	
EQ-5D			
Index score	0.92 ± 0.14	0.95 ± 0.10	<0.001
Good HRQoL	5585 (58.4)	6526 (68.2)	<0.001
Poor HRQoL	3977 (41.6)	3037 (31.8)	

## Data Availability

The dataset used and analyzed in the current study is available from the corresponding author on reasonable request. Raw data are available on the Korea Community Health Survey website (https://chs.kdca.go.kr/chs/index.do (accessed on 19 July 2022)).

## References

[1] World Health Organization (2021). Global Status Report on the Public Health Response to Dementia. https://www.who.int/publications/i/item/9789240033245.

[2] Ministry of Health and Welfare (2020). The 4th National Dementia Plan. https://ansim.nid.or.kr/community/pds_view.aspx?page=&BID=242.

[3] Alzheimer’s Association (2020). Alzheimer’s Disease Facts and Figures. Alzheimer’s Dement..

[4] Lee J.S., Kang M.J., Nam H.J., Kim Y.J., Lee O.J., Kim K.W. Korean Dementia Observatory 2019. https://www.nid.or.kr/info/dataroom_view.aspx?bid=209.

[5] Schwarzkopf L., Menn P., Kunz S., Holle R., Lauterberg J., Marx P., Mehlig H., Wunder S., Leidl R., Donath C. (2011). Costs of care for dementia patients in community setting: An analysis for mild and moderate disease stage. Value Health.

[6] Nordberg G., Wimo A., Jonsson L., Kareholt I., Sjolund B.M., Lagergren M., von Strauss E. (2007). Time use and costs of institutionalised elderly persons with or without dementia: Results from the Nordanstig cohort in the Kungsholmen Project—A population based study in Sweden. Int. J. Geriatr. Psychiatry.

[7] Gitlin L.N., Kales H.C., Lyketsos C.G. (2012). Nonpharmacologic management of behavioral symptoms in dementia. JAMA.

[8] Gaugler J.E., Kane R.L., Kane R.A. (2002). Family care for older adults with disabilities: Toward more targeted and interpretable research. Int. J. Aging Hum. Dev..

[9] Schulz R., O’Brien A.T., Bookwala J., Fleissner K. (1995). Psychiatric and physical morbidity effects of dementia caregiving: Prevalence, correlates, and causes. Gerontologist.

[10] Mausbach B.T., Chattillion E.A., Roepke S.K., Patterson T.L., Grant I. (2013). A comparison of psychosocial outcomes in elderly Alzheimer caregivers and noncaregivers. Am. J. Geriatr. Psychiatry.

[11] Bremer P., Cabrera E., Leino-Kilpi H., Lethin C., Saks K., Sutcliffe C., Soto M., Zwakhalen S.M., Wubker A., RightTimePlaceCare Consortium (2015). Informal dementia care: Consequences for caregivers’ health and health care use in 8 European countries. Health Policy.

[12] Pinquart M., Sorensen S. (2007). Correlates of physical health of informal caregivers: A meta-analysis. J. Gerontol. B Psychol. Sci. Soc. Sci..

[13] Ma M., Dorstyn D., Ward L., Prentice S. (2018). Alzheimers’ disease and caregiving: A meta-analytic review comparing the mental health of primary carers to controls. Aging Ment. Health.

[14] Sallim A.B., Sayampanathan A.A., Cuttilan A., Ho R. (2015). Prevalence of Mental Health Disorders among Caregivers of Patients with Alzheimer Disease. J. Am. Med. Dir. Assoc..

[15] Vitaliano P.P., Zhang J., Scanlan J.M. (2003). Is caregiving hazardous to one’s physical health? A meta-analysis. Psychol. Bull..

[16] van der Lee J., Bakker T.J., Duivenvoorden H.J., Droes R.M. (2014). Multivariate models of subjective caregiver burden in dementia: A systematic review. Ageing Res. Rev..

[17] de Vugt M.E., Jolles J., van Osch L., Stevens F., Aalten P., Lousberg R., Verhey F.R. (2006). Cognitive functioning in spousal caregivers of dementia patients: Findings from the prospective MAASBED study. Age Ageing.

[18] Palma K.A., Balardin J.B., Vedana G., de Lima A.I.I., Luz C., Schroder N., Quevedo J., Bromberg E. (2011). Emotional memory deficit and its psychophysiological correlate in family caregivers of patients with dementia. Alzheimer Dis. Assoc. Disord..

[19] Oken B.S., Fonareva I., Wahbeh H. (2011). Stress-related cognitive dysfunction in dementia caregivers. J. Geriatr. Psychiatry Neurol..

[20] Pertl M.M., Hannigan C., Brennan S., Robertson I.H., Lawlor B.A. (2017). Cognitive reserve and self-efficacy as moderators of the relationship between stress exposure and executive functioning among spousal dementia caregivers. Int. Psychogeriatr..

[21] Correa M.S., Vedovelli K., Giacobbo B.L., de Souza C.E., Ferrari P., de Lima A.I.I., Walz J.C., Kapczinski F., Bromberg E. (2015). Psychophysiological correlates of cognitive deficits in family caregivers of patients with Alzheimer Disease. Neuroscience.

[22] Correa M.S., Giacobbo B.L., Vedovelli K., Lima D.B., Ferrari P., Argimon I.I.L., Walz J.C., Bromberg E. (2016). Age Effects on Cognitive and Physiological Parameters in Familial Caregivers of Alzheimer’s Disease Patients. PLoS ONE.

[23] Correa M.S., de Lima D.B., Giacobbo B.L., Vedovelli K., Argimon I.I.L., Bromberg E. (2019). Mental health in familial caregivers of Alzheimer’s disease patients: Are the effects of chronic stress on cognition inevitable?. Stress.

[24] Fonareva I., Oken B.S. (2014). Physiological and functional consequences of caregiving for relatives with dementia. Int. Psychogeriatr..

[25] Chiu Y.C., Lee Y.N., Wang P.C., Chang T.H., Li C.L., Hsu W.C., Lee S.H. (2014). Family caregivers’ sleep disturbance and its associations with multilevel stressors when caring for patients with dementia. Aging Ment. Health.

[26] Peng H.L., Chang Y.P. (2013). Sleep disturbance in family caregivers of individuals with dementia: A review of the literature. Perspect. Psychiatr. Care.

[27] Beaudreau S.A., Spira A.P., Gray H.L., Depp C.A., Long J., Rothkopf M., Gallagher-Thompson D. (2008). The relationship between objectively measured sleep disturbance and dementia family caregiver distress and burden. J. Geriatr. Psychiatry Neurol..

[28] McCurry S.M., Logsdon R.G., Teri L., Vitiello M.V. (2007). Sleep disturbances in caregivers of persons with dementia: Contributing factors and treatment implications. Sleep Med. Rev..

[29] Gao C., Chapagain N.Y., Scullin M.K. (2019). Sleep Duration and Sleep Quality in Caregivers of Patients with Dementia: A Systematic Review and Meta-analysis. JAMA Netw. Open.

[30] McKibbin C.L., Ancoli-Israel S., Dimsdale J., Archuleta C., von Kanel R., Mills P., Patterson T.L., Grant I. (2005). Sleep in spousal caregivers of people with Alzheimer’s disease. Sleep.

[31] Simon M.A., Bueno A.M., Otero P., Blanco V., Vazquez F.L. (2019). Caregiver Burden and Sleep Quality in Dependent People’s Family Caregivers. J. Clin. Med..

[32] von Kanel R., Mausbach B.T., Ancoli-Israel S., Dimsdale J.E., Mills P.J., Patterson T.L., Ziegler M.G., Roepke S.K., Chattillion E.A., Allison M. (2012). Sleep in spousal Alzheimer caregivers: A longitudinal study with a focus on the effects of major patient transitions on sleep. Sleep.

[33] Song M.J., Kim J.H. (2021). Family Caregivers of People with Dementia Have Poor Sleep Quality: A Nationwide Population-Based Study. Int. J. Environ. Res. Public Health.

[34] The World Health Organization Quality of Life assessment (WHOQOL) (1995). Position paper from the World Health Organization. Soc. Sci. Med..

[35] Woods B., Spector A., Jones C., Orrell M., Davies S. (2005). Reminiscence therapy for dementia. Cochrane Database. Syst. Rev..

[36] Moniz-Cook E., Vernooij-Dassen M., Woods R., Verhey F., Chattat R., De Vugt M., Mountain G., O’Connell M., Harrison J., Vasse E. (2008). A European consensus on outcome measures for psychosocial intervention research in dementia care. Aging Ment. Health.

[37] Markowitz J.S., Gutterman E.M., Sadik K., Papadopoulos G. (2003). Health-related quality of life for caregivers of patients with Alzheimer disease. Alzheimer Dis. Assoc. Disord..

[38] Andreakou M.I., Papadopoulos A.A., Panagiotakos D.B., Niakas D. (2016). Assessment of Health-Related Quality of Life for Caregivers of Alzheimer’s Disease Patients. Int. J. Alzheimer’s Dis..

[39] Valimaki T.H., Martikainen J.A., Hongisto K., Vaatainen S., Sintonen H., Koivisto A.M. (2016). Impact of Alzheimer’s disease on the family caregiver’s long-term quality of life: Results from an ALSOVA follow-up study. Qual. Life Res..

[40] Goren A., Montgomery W., Kahle-Wrobleski K., Nakamura T., Ueda K. (2016). Impact of caring for persons with Alzheimer’s disease or dementia on caregivers’ health outcomes: Findings from a community based survey in Japan. BMC Geriatr..

[41] Laks J., Goren A., Duenas H., Novick D., Kahle-Wrobleski K. (2016). Caregiving for patients with Alzheimer’s disease or dementia and its association with psychiatric and clinical comorbidities and other health outcomes in Brazil. Int. J. Geriatr. Psychiatry.

[42] Bell C.M., Araki S.S., Neumann P.J. (2001). The association between caregiver burden and caregiver health-related quality of life in Alzheimer disease. Alzheimer Dis. Assoc. Disord..

[43] Serrano-Aguilar P.G., Lopez-Bastida J., Yanes-Lopez V. (2006). Impact on health-related quality of life and perceived burden of informal caregivers of individuals with Alzheimer’s disease. Neuroepidemiology.

[44] Bruvik F.K., Ulstein I.D., Ranhoff A.H., Engedal K. (2012). The quality of life of people with dementia and their family carers. Dement. Geriatr. Cogn. Disord..

[45] Froelich L., Llado A., Khandker R.K., Pedros M., Black C.M., Sanchez Diaz E.J., Chekani F., Ambegaonkar B. (2021). Quality of Life and Caregiver Burden of Alzheimer’s Disease Among Community Dwelling Patients in Europe: Variation by Disease Severity and Progression. J. Alzheimer’s Dis. Rep..

[46] Hvidsten L., Engedal K., Selbaek G., Wyller T.B., Saltyte Benth J., Bruvik F., Kersten H. (2020). Quality of life of family carers of persons with young-onset compared to late-onset dementia. Aging Ment. Health.

[47] Papastavrou E., Andreou P., Middleton N., Papacostas S., Georgiou I.K. (2014). Factors associated with quality of life among family members of patients with dementia in Cyprus. Int. Psychogeriatr..

[48] Valimaki T.H., Vehvilainen-Julkunen K.M., Pietila A.M., Pirttila T.A. (2009). Caregiver depression is associated with a low sense of coherence and health-related quality of life. Aging Ment. Health.

[49] Farina N., Page T.E., Daley S., Brown A., Bowling A., Basset T., Livingston G., Knapp M., Murray J., Banerjee S. (2017). Factors associated with the quality of life of family carers of people with dementia: A systematic review. Alzheimer’s Dement..

[50] Rabin R., de Charro F. (2001). EQ-5D: A measure of health status from the EuroQol Group. Ann. Med..

[51] Devlin N.J., Brooks R. (2017). EQ-5D and the EuroQol Group: Past, Present and Future. Appl. Health Econ. Health Policy.

[52] Kang Y.W., Ko Y.S., Kim Y.J., Sung K.M., Kim H.J., Choi H.Y., Sung C., Jeong E. (2015). Korea Community Health Survey Data Profiles. Osong Public Health Res. Perspect..

[53] Rim H., Kim H., Lee K., Chang S., Hovell M.F., Kim Y.T., Kim Y., Kang G., Tak Y., Im J. (2011). Validity of self-reported healthcare utilization data in the Community Health Survey in Korea. J. Korean Med. Sci..

[54] Lee Y.K., Nam H.S., Chuang L.H., Kim K.Y., Yang H.K., Kwon I.S., Kind P., Kweon S.S., Kim Y.T. (2009). South Korean time trade-off values for EQ-5D health states: Modeling with observed values for 101 health states. Value Health.

[55] Coups E.J., Ostroff J.S. (2005). A population-based estimate of the prevalence of behavioral risk factors among adult cancer survivors and noncancer controls. Prev. Med..

[56] Haskell W.L., Lee I.M., Pate R.R., Powell K.E., Blair S.N., Franklin B.A., Macera C.A., Heath G.W., Thompson P.D., Bauman A. (2007). Physical activity and public health: Updated recommendation for adults from the American College of Sports Medicine and the American Heart Association. Circulation.

[57] Taylor C.A., Bouldin E.D., McGuire L.C. (2018). Subjective Cognitive Decline Among Adults Aged ≥ 45 Years—United States, 2015–2016. MMWR. Morb. Mortal. Wkly. Rep..

[58] Kroenke K., Spitzer R.L., Williams J.B. (2001). The PHQ-9: Validity of a brief depression severity measure. J. Gen. Intern. Med..

[59] Han C., Jo S.A., Kwak J.H., Pae C.U., Steffens D., Jo I., Park M.H. (2008). Validation of the Patient Health Questionnaire-9 Korean version in the elderly population: The Ansan Geriatric study. Compr. Psychiatry.

[60] Buysse D.J., Reynolds C.F., Monk T.H., Berman S.R., Kupfer D.J. (1989). The Pittsburgh Sleep Quality Index: A new instrument for psychiatric practice and research. Psychiatry Res..

[61] Sohn S.I., Kim D.H., Lee M.Y., Cho Y.W. (2012). The reliability and validity of the Korean version of the Pittsburgh Sleep Quality Index. Sleep Breath..

[62] Mollayeva T., Thurairajah P., Burton K., Mollayeva S., Shapiro C.M., Colantonio A. (2016). The Pittsburgh sleep quality index as a screening tool for sleep dysfunction in clinical and non-clinical samples: A systematic review and meta-analysis. Sleep Med. Rev..

[63] Lee S.Y., Ju Y.J., Lee J.E., Kim Y.T., Hong S.C., Choi Y.J., Song M.K., Kim H.Y. (2020). Factors associated with poor sleep quality in the Korean general population: Providing information from the Korean version of the Pittsburgh Sleep Quality Index. J. Affect. Disord..

[64] Ware J.E., Gandek B. (1998). Overview of the SF-36 Health Survey and the International Quality of Life Assessment (IQOLA) Project. J. Clin. Epidemiol..

[65] Sintonen H. (2001). The 15D instrument of health-related quality of life: Properties and applications. Ann. Med..

[66] Kuo L.M., Huang H.L., Liang J., Kwok Y.T., Hsu W.C., Liu C.Y., Shyu Y.L. (2017). Trajectories of health-related quality of life among family caregivers of individuals with dementia: A home-based caregiver-training program matters. Geriatr. Nurs..

[67] Livingston G., Barber J., Rapaport P., Knapp M., Griffin M., King D., Livingston D., Mummery C., Walker Z., Hoe J. (2013). Clinical effectiveness of a manual based coping strategy programme (START, STrAtegies for RelaTives) in promoting the mental health of carers of family members with dementia: Pragmatic randomised controlled trial. BMJ.

[68] Kuo L.M., Huang H.L., Huang H.L., Liang J., Chiu Y.C., Chen S.T., Kwok Y.T., Hsu W.C., Shyu Y.I. (2013). A home-based training program improves Taiwanese family caregivers’ quality of life and decreases their risk for depression: A randomized controlled trial. Int. J. Geriatr. Psychiatry.

[69] Danucalov M.A., Kozasa E.H., Afonso R.F., Galduroz J.C., Leite J.R. (2017). Yoga and compassion meditation program improve quality of life and self-compassion in family caregivers of Alzheimer’s disease patients: A randomized controlled trial. Geriatr. Gerontol. Int..

[70] Graff M.J., Vernooij-Dassen M.J., Thijssen M., Dekker J., Hoefnagels W.H., Olderikkert M.G. (2007). Effects of community occupational therapy on quality of life, mood, and health status in dementia patients and their caregivers: A randomized controlled trial. J. Gerontol. A Biol. Sci. Med. Sci..

[71] Dooley N.R., Hinojosa J. (2004). Improving quality of life for persons with Alzheimer’s disease and their family caregivers: Brief occupational therapy intervention. Am. J. Occup. Ther..

[72] Belle S.H., Burgio L., Burns R., Coon D., Czaja S.J., Gallagher-Thompson D., Gitlin L.N., Klinger J., Koepke K.M., Lee C.C. (2006). Enhancing the quality of life of dementia caregivers from different ethnic or racial groups: A randomized, controlled trial. Ann. Intern. Med..

[73] Chien W.T., Lee I.Y. (2011). Randomized controlled trial of a dementia care programme for families of home-resided older people with dementia. J. Adv. Nurs..

[74] von Kanel R., Ancoli-Israel S., Dimsdale J.E., Mills P.J., Mausbach B.T., Ziegler M.G., Patterson T.L., Grant I. (2010). Sleep and biomarkers of atherosclerosis in elderly Alzheimer caregivers and controls. Gerontology.

[75] Marquez-Gonzalez M., Cabrera I., Losada A., Knight B.G. (2018). Attentional avoidant biases as mediators in the association between experiential avoidance and blood pressure in dementia family caregivers. Aging Ment. Health.

[76] Xu X.Y., Kwan R.Y.C., Leung A.Y.M. (2020). Factors associated with the risk of cardiovascular disease in family caregivers of people with dementia: A systematic review. J. Int. Med. Res..

[77] Contador I., Fernandez-Calvo B., Palenzuela D.L., Migueis S., Ramos F. (2012). Prediction of burden in family caregivers of patients with dementia: A perspective of optimism based on generalized expectancies of control. Aging Ment. Healt.

[78] Garcia-Alberca J.M., Lara J.P., Berthier M.L. (2011). Anxiety and depression in caregivers are associated with patient and caregiver characteristics in Alzheimer’s disease. Int. J. Psychiatry Med..

[79] Joling K.J., van Marwijk H.W., Veldhuijzen A.E., van der Horst H.E., Scheltens P., Smit F., van Hout H.P. (2015). The two-year incidence of depression and anxiety disorders in spousal caregivers of persons with dementia: Who is at the greatest risk?. Am. J. Geriatr. Psychiatry.

[80] Logsdon R.G., Gibbons L.E., McCurry S.M., Teri L. (2002). Assessing quality of life in older adults with cognitive impairment. Psychosom. Med..

[81] Thorgrimsen L., Selwood A., Spector A., Royan L., de Madariaga Lopez M., Woods R.T., Orrell M. (2003). Whose quality of life is it anyway? The validity and reliability of the Quality of Life-Alzheimer’s Disease (QoL-AD) scale. Alzheimer Dis. Assoc. Disord..

[82] Hounsome N., Orrell M., Edwards R.T. (2011). EQ-5D as a quality of life measure in people with dementia and their carers: Evidence and key issues. Value Health.

[83] Coucill W., Bryan S., Bentham P., Buckley A., Laight A. (2001). EQ-5D in patients with dementia: An investigation of inter-rater agreement. Med. Care.

[84] Karlawish J.H., Zbrozek A., Kinosian B., Gregory A., Ferguson A., Glick H.A. (2008). Preference-based quality of life in patients with Alzheimer’s disease. Alzheimer’s Dement..

[85] Riedijk S.R., De Vugt M.E., Duivenvoorden H.J., Niermeijer M.F., Van Swieten J.C., Verhey F.R., Tibben A. (2006). Caregiver burden, health-related quality of life and coping in dementia caregivers: A comparison of frontotemporal dementia and Alzheimer’s disease. Dement. Geriatr. Cogn. Disord..

[86] Mioshi E., Foxe D., Leslie F., Savage S., Hsieh S., Miller L., Hodges J.R., Piguet O. (2013). The impact of dementia severity on caregiver burden in frontotemporal dementia and Alzheimer disease. Alzheimer Dis. Assoc. Disord..

[87] Uflacker A., Edmondson M.C., Onyike C.U., Appleby B.S. (2016). Caregiver burden in atypical dementias: Comparing frontotemporal dementia, Creutzfeldt-Jakob disease, and Alzheimer’s disease. Int. Psychogeriatr..

[88] Liu S., Jin Y., Shi Z., Huo Y.R., Guan Y., Liu M., Liu S., Ji Y. (2017). The effects of behavioral and psychological symptoms on caregiver burden in frontotemporal dementia, Lewy body dementia, and Alzheimer’s disease: Clinical experience in China. Aging Ment. Health.

[89] Wiegelmann H., Speller S., Verhaert L.M., Schirra-Weirich L., Wolf-Ostermann K. (2021). Psychosocial interventions to support the mental health of informal caregivers of persons living with dementia—A systematic literature review. BMC Geriatr..

